# Measuring fragmentation of ambulatory care in a tripartite healthcare system

**DOI:** 10.1186/1472-6963-13-176

**Published:** 2013-05-15

**Authors:** Su Liu, Philip C Yeung

**Affiliations:** 1 Chinese University of Hong Kong, Shatin, NT, Hong Kong; 2 5/F, School of Public Health, Prince of Wales Hospital, Shatin, NT, Hong Kong; 3 4/F, School of Public Health, Prince of Wales Hospital, Shatin, NT, Hong Kong

**Keywords:** Fragmentation, Continuity of care, Ambulatory care, Chinese medicine, Primary care

## Abstract

**Background:**

Hong Kong has a tripartite healthcare system, where western medicine provided in both public and private sectors coexist with Chinese medicine practice. The purpose of this study is to measure fragmentation of ambulatory care experienced by the non-institutionalized population aged 15 and over in such a tripartite system, thus shed light on the ongoing primary care reform.

**Methods:**

This is a cross-sectional secondary data analysis using the Thematic Household Survey, which was conducted by the Hong Kong Census and Statistics Department during November 2009 to February 2010 to collect territory-wide health-related information. Among 18,226 individuals with two or more ambulatory visits during the past 12 months before interview, we grouped each visit into one of the three care segments—public western, private western and Chinese medicine. Two individual-level measures were used to quantify longitudinal fragmentation of care across segments over the one-year period: Most Frequent Provider Continuity Index (MFPC) and Fragmentation of Care Index (FCI). Both are analyzed for distribution and subgroup comparison. A Tobit model was used to further examine the determinants of fragmentation.

**Results:**

More than a quarter of individuals sought care in two or all three segments, with an average MFPC of 65% and FCI of 0.528. Being older, female, married, unemployed, uninsured, or born in mainland China, with lower education, lower income, higher number of chronic conditions or poorer health were found to have experienced higher fragmentation of care. We also found that, fragmentation of care increased with the total number of ambulatory care visits and it varied significantly depending on what segment the individual chose to visit most frequently—those chose private western clinics had lower FCI, compared with those chose public western or Chinese medicine as the most frequently visited segment.

**Conclusions:**

Even measured at healthcare segment level, people in Hong Kong experienced modest fragmentation of care. Individuals’ health beliefs—as a result of the persistent habitual tendency and latitude incentivized by the system—may be behind the fragmented care we saw. Efforts are needed to alter health beliefs, targeting subgroups of vulnerable population, and create environments that promote better coordinated primary care.

## Background

Primary health care is important for population health as it is the frontline interface between the population and the healthcare delivery system [1]. Effective primary care can lead to better health outcomes, lower costs and greater equity in health, with robust evidence across a wide variety of studies worldwide [2,3]. As a defining characteristic of primary care [4], continuity of care (i.e. the relationship between patients and physicians that goes beyond any specific episode of illness or disease) [5] has been shown to lead to better patient outcomes, such as fewer emergency department visits [6,7], more preventive care [8,9], decreased hospital admissions [10,11], better chronic disease control [12,13], and less intensive care unit use [14]. On the other hand, the lack of continuity, often known as fragmented care, has been linked with negative outcomes, especially among those with chronic diseases [15-18]. Given the evidence and the enormous access, cost and quality challenges healthcare systems around the world face today, the World Health Organization (WHO) renewed the call for primary care reform after 30 years of the Declaration of Alma-Ata [1].

Facing the same challenges and responding to WHO’s call, the Hong Kong (HK) government established a Primary Care Office under the Department of Health in September 2010 to lead its own primary care reform [19]. As an ex-British-colony, the HK healthcare system has evolved from a tax-funded model, with a traditional focus on hospital care. However, the concept of primary care and having a regular doctor has neither been widely appreciated by patients nor practiced among providers. As a result, people seek ambulatory care from all types of generalists and specialists in any public or private clinics they may have access to. For the purpose of this study, we viewed the delivery of primary care in Hong Kong as a tripartite system. Western medicine still plays a major role with the co-existence of public and private sectors and a roughly 3-to-7 split of market share, correspondingly [20]. However, since over 95% of the population is ethnic Chinese, the existence of Traditional Chinese Medicine (TCM) cannot be undermined especially after the formal recognition of TCM practitioners since 2002 [21]. Except a few “public” TCM clinics which are partially funded by the Government but operated by the local non-government organizations, most TCM services are provided by private doctors [21]. Integrated Chinese and western medicine service only exist in limited number of pilot programs for outpatient treatment of specific diseases, rehabilitation and palliative care. Given the parallel nature, we considered Chinese medicine (regardless of public or private) as the third segment in addition to the public and private western medicine split.

The three segments are widely recognized by the public as options for ambulatory care, but operate rather independently. Such distinction is reflected not only in patient experience but also in the financing mechanism as well as Government policy. From patients’ perspective, the waiting time in public western clinics has long been criticized, but their costs are just 1/4 of private general western practitioners and 1/9 of private western specialists [22]. It is also a common belief that Chinese medicine emphasizes personalized care, balance of bodily factors and disease prevention [21], which is different from western medicine. Based on these real and perceived differences across segments, people can choose from 122 public western clinics, over 3,700 private western clinics [20], and over 9,000 Chinese medicine practitioners, mostly in solo-practice [23]. In terms of financing mechanism, Government general tax revenue funds the majority (around 95%) of public sector services (mostly western medicine), while individuals’ out-of-pocket spending funds the majority (around 70%) of the private sector services (both western and Chinese medicines) [20]. Private insurance is purchased voluntarily and many insurance policies do not cover visits to the Chinese medicine practitioners. Government policy has traditionally focused on western medicine and the public sector. For example, within the public western medicine segment, information sharing between a large majority of public clinics was made possible by the Clinical Management System (CMS) developed by Hospital Authority since 2000. Such within-segment information continuity, however, does not exist in the other two segments or across segments. Recently, the Hong Kong Government has started paying more attention to the further development of the private healthcare sector and Chinese medicine, as well as integration across the three segments [24].

With such backdrop, the main objective of this paper is to measure fragmentation of care across the three segments of primary care system in Hong Kong, thus shed light on the future direction of the ongoing primary care reform. Although local population’s utilization prevalence and doctor-shopping behavior have been studied previously, our analysis extends the knowledge about individual-level experience of fragmented care by filling a few gaps. First, instead of providing overall utilization statistics, such as number of people or visits to different segments of care [25-27], we looked at individual-level utilization patterns, which should more directly reflect experience in continuity of care. Second, changing of doctors without professional referral in a single illness episode (often referred as “doctor-shopping”) has long been recognized as one of the substantial contributing factor to the high levels of ambulatory care utilization in Hong Kong [28]. However, by definition, these studies only looked at switching doctors within a single illness episode, and almost all focused only among patients attending public western medicine clinics [29,30]. Our study examined utilization beyond a single episode over a longer time period for all adult population in Hong Kong. Third, utilization of Chinese medicine has often been studied separately, as opposed to being considered in parallel with the public and private provision of western medicine services [21,31]. With this paper, we intend to define and analyze measures of longitudinal fragmentation of care in the local context, for the first time paying attention to all three segments of the tripartite system. Results will help clarify the extent of fragmentation in the ambulatory care delivery system and identify influential factors, thus paving the way to form better primary care development strategies that support creative interaction between different parts of the system, not only in Hong Kong, but also elsewhere that face similar challenges.

## Methods

### Data source and study population

Since 1999, the Census and Statistics Department of the Hong Kong Special Administrative Region Government conducts a series of territory-wide cross-sectional Thematic Household Survey (THS) to collect statistical data on different social topics periodically. These surveys are governed by the Census and Statistics Ordinance and subsidiary legislation, which provide strict safeguards on the ethics and confidentiality of data. Our study was based on the round of THS conducted during November 2009 to February 2010 to collect information on health-related topics, including the health status, insurance benefits and healthcare utilization of Hong Kong residents. The face-to-face survey included 10,028 households representing land-based non-institutional population of Hong Kong, with a response rate of 75 percent [32]. The Food and Health Bureau of the Hong Kong SAR Government granted us access to the health-related data items from this survey for research purpose.

The survey question of most interest to us was “In the past 12 months, have you had a consultation with a doctor either inside or outside Hong Kong?” and if “yes”, “which of the following types of clinics have you visited? For every type, approximately how many times have you visited?” A total of 25 different types of clinics were listed in the original questionnaire, without identification of specific clinic or provider names. To meet our purpose of assessing individuals’ experience of seeking ambulatory care across the tripartite system, we grouped the 25 different types of clinics into three categories—western medicine clinics in the public sector (operated by either Hospital Authority or Department of Health), private western medicine clinics (including outpatient departments of private hospitals), and Chinese medicine clinics (mostly private). Thus, each individual’s ambulatory care during the year can be characterized by concentration or dispersion in one or more segments.

We selected persons aged 15 or above with 2 or more visits in the year as our study population. Children under age 15 were excluded, as they were not asked of health condition and other related questions. Also excluded were people with zero or only one visit during the year, because they had no “chance” to experience different segments of care. We also disregarded visits to Accident and Emergency Departments or to clinics outside of Hong Kong.

### Outcome measures of fragmentation

The primary outcome was the longitudinal fragmentation of ambulatory visits across the three major care segments over a 12-month period. There is an existing body of work that defines and measures the opposite side of the same coin, that is, the continuity of care [33]. Several similarities and differences between our study and this literature deserve mentioning. First, like the majority of existing measures of continuity of care, our measures related to visit patterns over time, representing the longitudinal nature of continuity, as opposed to informational or interpersonal continuity of care, which are much harder to measure. Second, most of previous studies looked at continuity of care across different physicians or clinics, whereas ours used a broader definition, that is, the major care segment. It might be considered as a more crude measure; therefore, a person experiencing zero fragmentation in our study does not imply perfect continuity of care, as he might be seeing different physicians within the same segment. On the other hand, a person experiencing medium level fragmentation in our study may signal even more fragmented care. Third, continuity of care has usually been treated as a process that may lead to better patient outcomes, and less as an outcome in its own right [34]. Here we regarded it as an outcome and a dependent variable, which we tried to identify factors to explain and improve.

Two measures developed from the existing literature were used to quantify individual-level fragmentation. One was the Most Frequent Provider Continuity Index (MFPC), which required first identifying the segment with the highest frequency of visits during the year, then calculating the fraction of total visits to that particular segment [35,36]. The higher the fraction, the more concentrated visits are to the dominant segment. The other measure was the Fragmentation of Care Index (FCI), which was based on the total number of visits, different segments visited, and the proportion of visits to each segment. We calculated the FCI—developed from the Continuity of Care Index (COC)—as follows [37,38]:

$$\mathrm{FCI}=1-\mathrm{COC}=1-\frac{\sum_{i=1}^{s}n_{i}^{2}-n}{n\left(n-1\right)}=\frac{n^{2}-\sum_{i=1}^{s}n_{i}^{2}}{n\left(n-1\right)}$$

where n indicates total number of visits; n_i_, the number of visits to segment i; and s, the number of segments visited. The FCI ranges from 0 (all visits to the same segment) to 1 (each visit to a different segment). Higher FCI value implies more fragmented care. To give an example, if a person had six doctor consultations during the year, one in a public western medicine clinic, two in the private western medicine segment and three visits to Chinese medicine clinics; his MFPC would be 50% and FCI would be 0.73.

By definition, the two measures were highly correlated, but as shown later, they depicted different aspects of individuals’ experience of care. MFPC indicated intuitively how concentrated individuals’ care was in the most seen segment, but didn’t offer any insight to the residual visits dispersed in other segments. FCI, though less intuitive, offered a more complete picture by taking all visited segments and level of dispersion into consideration.

### Statistical analysis

MFPC and FCI were calculated at the individual level and summarized among all study population and by different combination of segments visited as well as socio-demographics and other health and healthcare related factors, such as number of chronic conditions, insurance coverage, etc. Sub-group differences were evaluated using one-way ANOVA. In addition to binary statistics, we also conducted a multivariate analysis of FCI as the dependent variable, to estimate relative importance of individual characteristics and healthcare utilization patterns in affecting the level of fragmentation experienced. As shown next, our study population saw approximately normally distributed positive FCI values, plus a large number of zeros. This data censoring can lead to biased coefficients if estimated using ordinary least squares linear regression. Instead, we used Tobit regression to correct for the left censoring of FCI [39-41]. A reduced-form model with predisposing, enabling and need factors as covariates, following Anderson’s behavioral model, was estimated by STATA [42,43].

## Results

**Figure 1:**
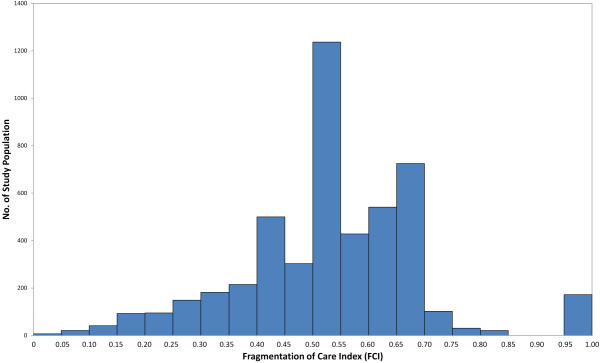
**Distribution of Fragmentation of Care Index (FCI) for the Study Population Excluding Zero (n = 4,863).** Figure 1 showed distribution of FCI among 4,863 individuals who had non-zero FCI values, with the mean FCI of 0.528 (SD = 0.160, median = 0.533).

Table 1 described our study population and summarized FCI by key characteristics. We identified 18,226 individuals aged 15 or above, with 2 or more ambulatory visits during the year. On average, they made slightly more than six ambulatory care visits over the 1-year period and the mean FCI was 0.141. This reflected that a large majority (73%) of them stayed in a single segment—10,030 continuously visited clinics within the private western medicine segment; 3,036, public western medicine; and 297, Chinese medicine. Each of them would have a MFPC of 100% and an FCI of zero. For the remaining 4,863 individuals (27%), visits were spread between two or all three segments, with the mean FCI of 0.528 (SD = 0.160, median = 0.533), as shown in the distribution (Figure 1). More specifically, we found using a combination of public and private western medicine services to be the most popular visit pattern, followed by the combination of private western and Chinese medicine services (Table 2). Visits were made to all three segments by 443 individuals and the combination of public western and Chinese medicine services was the least popular pattern.

**Table 1:** Characteristics of the Study Population and Comparison of Mean Fragmentation of Care Index (FCI)

	**N (%)**	**FCI Mean (SD)**	***p*-value**
Total	18,226 (100%)	0.141 (0.248)	
Age			<0.0001
15-24	2,380 (13.1%)	0.096 (0.233)	
25-34	2,792 (15.3%)	0.092 (0.215)	
35-44	3,100 (17.0%)	0.117 (0.234)	
45-54	3,781 (20.7%)	0.146 (0.253)	
55-64	2,768 (15.2%)	0.181 (0.263)	
65+	3,405 (18.7%)	0.194 (0.260)	
Gender			<0.0001
Male	8,362 (45.9%)	0.127 (0.242)	
Female	9,864 (54.1%)	0.152 (0.252)	
Marital status			<0.0001
Single	5,373 (29.5%)	0.096 (0.223)	
Married	10,759 (59.0%)	0.153 (0.253)	
Divorced/Separated	617 (3.4%)	0.179 (0.263)	
Widowed	1,477 (8.1%)	0.201 (0.261)	
Education			<0.0001
Below primary	1,208 (6.6%)	0.190 (0.257)	
Primary	3,765 (20.7%)	0.181 (0.261)	
Secondary	10,361 (56.8%)	0.127 (0.243)	
Tertiary or above	2,892 (15.9%)	0.119 (0.235)	
Birthplace			<0.0001
Hong Kong	11,971 (65.7%)	0.124 (0.237)	
Mainland China, Macau or Taiwan	5,757 (31.6%)	0.180 (0.267)	
Overseas	498 (2.7%)	0.094 (0.212)	
Monthly household income (in HKD)			<0.0001
<$ 9,999	4,034 (22.1%)	0.173 (0.262)	
$10,000-19,000	5,048 (27.7%)	0.142 (0.251)	
$20,000-29,000	3,646 (20.0%)	0.130 (0.241)	
$30,000-39,000	2,162 (11.9%)	0.129 (0.239)	
$40,000 or above	3,336 (18.3%)	0.120 (0.234)	
Employment			<0.0001
Currently employed	9,624 (52.8%)	0.121 (0.238)	
Not employed	8,602 (47.2%)	0.163 (0.256)	
Received medical benefits from employer or bought medical insurance			<0.0001
Yes	8,181 (44.9%)	0.124 (0.236)	
No	10,045 (55.1%)	0.154 (0.256)	
Number of chronic conditions			<0.0001
0	11,331 (62.2%)	0.102 (0.231)	
1	3,941 (21.6%)	0.187 (0.261)	
2	1,722 (9.4%)	0.212 (0.255)	
3 or more	1,232 (6.8%)	0.247 (0.262)	
Self-reported health status			<0.0001
Excellent/Very good/Good	12,370 (67.9%)	0.120 (0.239)	
Fair/Poor	5,856 (32.1%)	0.185 (0.259)	
Total number of ambulatory care visits			<0.0001
2	4,231 (23.2%)	0.040 (0.197)	
3	3,208 (17.6%)	0.065 (0.199)	
4	2,604 (14.3%)	0.113 (0.239)	
5	1,534 (8.4%)	0.158 (0.252)	
6	1,683 (9.2%)	0.174 (0.257)	
7 or more	4,966 (27.2%)	0.273 (0.253)	
Most frequently visited segment			<0.0001
Public western	4,739 (26.0%)	0.176 (0.246)	
Private western	11,507 (63.1%)	0.064 (0.174)	
Chinese medicine	1,053 (5.8%)	0.332 (0.246)	
Equal visits to 2 or more segments	927 (5.1%)	0.696 (0.156)	

**Table 2:** Average Most Frequent Provider Continuity Index (MFPC) for Hong Kong residents, aged 15 or above, with 2 or more ambulatory care visits in 2 or more segments over a one-year period around 2009, by visit patterns and the most frequently visited segment

**Visit patterns**	**Most frequently visited segment**				**People with equal visits to 2 or more segments**
	**Public Western**	**Private Western**	**Chinese Medicine**	**Overall**	
	**MFPC Mean**			***(number of individuals, column %)***	
	***(number of individuals, row %)***				
Public Western + Private Western	70.14%	69.05%	N/A	69.72%	50%
	*(1,457, 61%)*	*(915, 39%)*		*(2,372, 60%)*	*(n = 603)*
Public Western + Chinese Medicine	69.60%	N/A	73.37%	71.33%	50%
	*(106, 54%)*		*(90, 46%)*	*(196, 5%)*	*(n = 34)*
Private Western + Chinese Medicine	N/A	69.66%	71.87%	70.79%	50%
		*(452, 49%)*	*(473, 51%)*	*(925, 24%)*	*(n = 197)*
Public Western + Private Western + Chinese Medicine	53.53%	55.42%	60.68%	57.12%	33-40%
	*(140, 32%)*	*(110, 25%)*	*(193, 44%)*	*(443, 11%)*	*(n = 93)*

Table 2 further identified the most frequently visited segment among individuals with each of the aforementioned visit patterns, and presented the average MFPC. For individuals with visits to two segments, the average MFPC hovered around 70%, regardless of specific pattern or most frequently visited segment. It dropped to just-over-50% for individuals with visits to all three segments, indicating roughly half of consultations happened in the most frequently visited segment and the other half split between the other two segments. Chinese medicine, often considered as complementary or alternative, was reflected so from the aggregated prevalence data. However, when looking at individuals using Chinese medicine in combination with other segments, it’s interesting to see many made the most frequent visits to Chinese medicine clinics; and on average, they had higher MFPC, signaling more concentrated care, compared with those made the most frequent visits to public or private western medicine segments.

To further explore the relationship between the fragmentation of care and its various predictors, we conducted bivariate and multivariate analysis, results of which were presented in Tables 1 and 3, respectively. We found statistically significant differences in the mean FCI across subgroups of population defined by predisposing characteristics (age, gender, marital status, education, and birth place), enabling resources (income, employment status and insurance coverage), and need factors (number of chronic conditions and self-reported health status). Being older, female, married, unemployed, uninsured, or born in mainland China, with lower education attainment, lower income, higher number of chronic conditions or poorer health status were found to have experienced higher fragmentation of care (Table 1). We also found that, fragmentation of care increased with the total number of ambulatory care visits and it varied significantly depending on what segment the individual chose to visit most frequently—those chose private western clinics had lower FCI, compared with those chose public western or Chinese medicine as the most frequently visited segment.

**Table 3:** Results of the Tobit Regression of Fragmentation of Care Index

	**Coefficient**	**95% Confidence interval**	***p*-value**
Age *(reference = age 15-24)*			
25-34	−0.054*	(-0.103 – -0.004)	0.035
35-44	−0.053	(-0.107 – 0.001)	0.053
45-54	−0.010	(-0.064 – 0.043)	0.711
55-64	0.038	(-0.018 – 0.094)	0.183
65+	0.009	(-0.051 – 0.070)	0.764
Female	0.070***	(0.047 – 0.093)	<0.001
Marital status *(reference = single)*			
Married	0.086***	(0.050 – 0.122)	<0.001
Divorced/Separated	0.047	(-0.016 – 0.111)	0.142
Widowed	0.096***	(0.043 – 0.148)	<0.001
Education *(reference = below primary)*			
Primary	0.055*	(0.011 – 0.099)	0.015
Secondary	0.065**	(0.018 – 0.112)	0.007
Tertiary or above	0.069*	(0.013 – 0.126)	0.016
Birthplace *(reference = born in HK)*			
Mainland China, Macau or Taiwan	0.042**	(0.017 – 0.066)	0.001
Overseas	−0.161***	(-0.232 – -0.089)	<0.001
Household size	−0.007	(-0.017 – 0.002)	0.137
Monthly household income *(reference = below $10,000)*			
$10,000-19,000	0.024	(-0.008 – 0.056)	0.144
$20,000-29,000	0.035	(-0.001 – 0.072)	0.058
$30,000-39,000	0.031	(-0.012 – 0.075)	0.160
$40,000 or above	<-0.001	(-0.041 – 0.041)	1.000
Currently employed	0.010	(-0.018 – 0.038)	0.480
Received medical benefits from employer or bought medical insurance	0.017	(-0.009 – 0.042)	0.201
Number of chronic conditions	0.049***	(0.038 – 0.060)	<0.001
Self-reported fair/poor health status	0.024	(0.000 – 0.048)	0.055
Quitted smoking	0.051*	(0.006 – 0.097)	0.028
Quitted drinking	0.057*	(0.003 – 0.110)	0.037
Did vigorous physical activities weekly	0.064***	(0.036 – 0.092)	<0.001
Total number of ambulatory care visits	0.014***	(0.013 – 0.016)	<0.001
Most frequently visited segment *(reference = public western)*			
Private western	−0.302***	(-0.330 – -0.275)	<0.001
Chinese medicine	0.280***	(0.238 – 0.322)	<0.001
Equal visits to 2 or more segments	0.888***	(0.847 – 0.929)	<0.001
Constant	−0.523***	(-0.599 – -0.447)	<0.001

Note: * Significant at *p* < 0.05, ** *p* < 0.01, *** *p* < 0.001. N = 18,226, Pseudo R^2^ = 0.256.

The Tobit regression confirmed some of the above relationships, but not all (Table 3). In addition to the aforementioned predictors, we also included household size to adjust the categorical household income variable, as well as three additional health behavior variables—if one had quitted smoking, quitted drinking, or done vigorous physical activities weekly—as proxies for proactive attitude towards health. The associations between the enabling factors and fragmentation of care were no longer statistically significant, after controlling for all the covariates. Most of the predisposing characteristics were still highly predictive of FCI values, especially gender, marital status, and birthplace. We saw diminishing influence of age and positive relationship between education and fragmentation of care in the regression analysis. In terms of actual or perceived healthcare need, those with more chronic conditions, more proactive health attitude and higher number of visits were more likely to have increased FCI too. The most frequently visited segment was by far the strongest predictor of fragmentation of care: compared to individuals who made most frequent visits to the public western medicine segment, those to the private western medicine segment had lower FCI, whereas those to the Chinese medicine segment had greater FCI.

## Discussion

We measured and analyzed fragmentation of ambulatory care for adult population in Hong Kong, where the primary care system is still under-developed and people can use any one segment or combination of the tripartite system—public western, private western and Chinese medicine—to access care. We found that, despite a large majority of people sticking to one segment for care during the year, the average FCI for the remaining quarter of population was 0.53, signaling modest fragmentation of care. Considering our measurement was done at the broad level of care segment, as opposed to individual doctors or clinics, people’s real experience from one episode of care to the next (or even within single episode) was likely to be much more fragmented than what’s shown here. Underestimation could also result from recall bias or under-reporting of service utilization in the original household interviews. Similar to a previous study of THS [25], we found that 8% of visits to public general outpatient clinics and even higher percent of visits to specialty clinics—according to the government administrative records—were not recalled by THS respondents. How exactly this would affect fragmentation was hard to predict, but based on our finding that people with more visits tend to experience more fragmented care, it might exacerbate the underestimation.

In the THS questionnaire, there was a question asking “Do you have a ‘regular/usually visited doctor’?” Only a quarter of our study population answered “yes”. Furthermore, these people were asked “where is your regular/usually visited doctor working in?” When comparing their responses to the most frequently visited segment (used in calculating the MFPC), we found roughly 20% un-matched cases. The lack of regular source of care and the confusing identification of who was the regular doctor again echoed the fragmented care found in our analysis.

Though our choice of studying fragmentation of care at the segment level was partly due to the limited availability of data, it was also a decision made based on the current stage of primary care development in Hong Kong. As mentioned in the background, information sharing across segments is still rare. The Hong Kong Government has been actively pursuing electronic health records sharing between the public and private sectors, but the result to date is not optimistic [44]. Many private doctors still keep patient records in paper form, especially Chinese medicine practitioners. Without electronic records, continuity of care across segments would be impossible. Our analysis also showed that many users of Chinese medicine services did not just consider it as complementary; instead, they depend on it almost as the primary care provider, as indicated by the dominant frequency of visits (higher MFPC), which could be a reflection of more personalized service or supplier-induced demand. However, we also found that the frequent users of Chinese medicine segments were the most likely to seek care across the other two segments as well (higher FCI). Both findings underscored the importance of incorporating Chinese medicine in the formal care system. Similar implications could be drawn for other healthcare systems where alternative medicine plays a noticeable role.

In addition to defining continuity at the broad segment level and data limitations mentioned above, other study limitations warrant consideration. First, unlike many previous studies that took continuity of care as a process measure and examined its association with health outcomes, our study treated it as an outcome and tried to explain its variation using a behavioral model. Although the word “fragmentation” seemed to have a built-in negative meaning to it, the model itself is non-normative. We didn’t have the data to assess if higher FCI as measured would lead to worse health or inefficiency, though previous evidence seemed to suggest so [15-18]. Seeking care across different segments sometimes helps to achieve better health outcomes. For example, cancer patients under chemotherapy often find Chinese medicine effective in mitigating pain, and people with multiple chronic conditions may be referred to different specialists, some may not be available within a single segment. However, the high rates of fragmentation we found for many individuals, the lack of information continuity across segments and the inherited silos in each segment’s development all raise serious concerns for patient safety as well as quality and efficiency of care. Second, using cross-sectional data, we tried to measure association of identified population characteristics with their experience of fragmented care. Inevitably there might be other determinants we had missed, particularly the supply-side or system factors. For example, some individuals would have preferred staying within the public western medicine segment if not because of the long travel or waiting times. Even for those factors included in our analysis, we were not able to draw causal conclusions, as some variables, for example, the total number of visits, could be endogenous.

## Conclusions

Despite the above limitations, our study shed light on how to improve continuity of care in Hong Kong and elsewhere that have a mixed healthcare system and underdeveloped primary care culture. We did not find strong association between fragmented care and the under-privileged. On the contrary, the enabling factors, such as income and insurance coverage, did not affect FCI significantly, after we controlled for other covariates. People with proactive attitude towards health (signaled by smoking or drinking cessation and exercise) experienced higher fragmentation, whereas people born overseas had lower FCI. The evidence suggests individuals’ health beliefs—as a result of the persistent habitual tendency and latitude incentivized by the system—perhaps drove the current utilization pattern we saw. Albeit the difficulty, health beliefs are mutable. This would require a powerful public campaign on the concept of primary care and the benefit of having a regular physician. Targeting subgroups, such as those with chronic conditions, might be more feasible and beneficial, as they were experiencing higher level of fragmentation yet more vulnerable to adverse effects of fragmented care. As long as the different provider segments co-exist, a certain level of fragmentation is unavoidable and perhaps even legitimate for reasons mentioned above. The goal is not to constrain people within a single segment for care. Instead of acting on the parts without appreciating their relation to the whole [45,46], policymakers ought to recognize the different roles each segment plays, and develop system-level changes (e.g. information technology improvement, financing reform, etc) across segments, thus creating an environment that encourages people to have a regular source of care and coordinates cross-segment care when needed.

## Competing interests

The authors declare that they have no competing interests.

## Authors’ contributions

SL conceived of and designed the study. PCY participated in the design of the study and performed the statistical analysis. Both interpreted the findings and drafted the manuscript. Both authors read and approved the final manuscript.

## Pre-publication history

The pre-publication history for this paper can be accessed here:

http://www.biomedcentral.com/1472-6963/13/176/prepub
